# A Bacteria-Based Self-Healing Cementitious Composite for Application in Low-Temperature Marine Environments

**DOI:** 10.3390/biomimetics2030013

**Published:** 2017-07-14

**Authors:** Damian Palin, Virginie Wiktor, Henk M. Jonkers

**Affiliations:** Microlab, Section of Materials and Environment, Faculty of Civil Engineering and Geosciences, Delft University of Technology, 2628 CN Delft, The Netherlands; v.a.c.wiktor@tudelft.nl (V.W.); h.m.Jonkers@tudelft.nl (H.M.J.)

**Keywords:** self-healing concrete, bacteria-actuated, marine, low-temperature, organic–inorganic composite

## Abstract

The current paper presents a bacteria-based self-healing cementitious composite for application in low-temperature marine environments. The composite was tested for its crack-healing capacity through crack water permeability measurements, and strength development through compression testing. The composite displayed an excellent crack-healing capacity, reducing the permeability of cracks 0.4 mm wide by 95%, and cracks 0.6 mm wide by 93% following 56 days of submersion in artificial seawater at 8 °C. Healing of the cracks was attributed to autogenous precipitation, autonomous bead swelling, magnesium-based mineral precipitation, and bacteria-induced calcium-based mineral precipitation in and on the surface of the bacteria-based beads. Mortar specimens incorporated with beads did, however, exhibit lower compressive strengths than plain mortar specimens. This study is the first to present a bacteria-based self-healing cementitious composite for application in low-temperature marine environments, while the formation of a bacteria-actuated organic–inorganic composite healing material represents an exciting avenue for self-healing concrete research.

## 1. Introduction

Traditional engineered materials are static and unresponsive in nature. Biological materials, in contrast, have evolved to respond and adapt to environmental and physiological stimuli. Self-healing is an outstanding biological material response whereby damage can be autonomously healed, restoring functional performance. Bone, for example, is able to form a blood clot around a fracture site. This clot becomes a cartilaginous callus, which later undergoes revascularization and calcification, and is finally remodeled, restoring bone functionality [1]. Biological materials, such as bone, have inspired a revolution in materials design and the development of a new class of smart self-healing materials. These engineered self-healing materials promise longer functional service lives with tremendous associated economic benefits [2,3].

**Figure 1:**
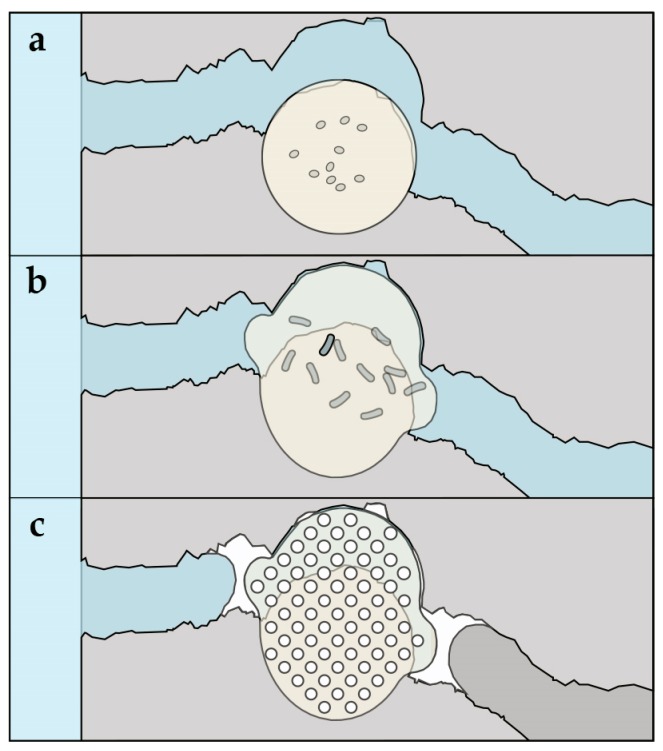
Schematic diagram illustrating the proposed healing mechanism: (**a**) in the event of cracking and water ingress; (**b**) the bacteria-based beads incorporated in the composite will swell, this swelling will clog the cracks, and concomitantly “free up” the bacteria, yeast extract and magnesium acetate contained in the beads; (**c**) the magnesium will precipitate as magnesium-based minerals, the spores will germinate as a result of being exposed to the solubilized yeast extract, and metabolize the acetate, inducing calcium-based mineral precipitation in and on the surface of the beads, healing the crack.

An innovative self-healing materials approach is one whereby bacteria immobilized in concrete are able to form a crack-healing precipitate [4,5,6,7,8,9,10,11,12,13,14]. Jonkers first introduced a bacteria-based agent—consisting of bacterial spores and an organic mineral precursor compound—for achieving bacteria-based self-healing concrete [4]. The bacteria spores activated by crack-induced water ingress germinate into active vegetative cells, which are able to convert mineral precursor compounds to calcium carbonate. Calcium carbonate, if precipitated in the cracks of steel-reinforced concrete, can result in the materials regain of functional water tightness and reduce, for example, its susceptibility to chloride ingress and reinforcement corrosion. Despite much of the world’s marine infrastructure being located in cool climatic zones (annual average temperature <10 °C, and average summer temperature generally <20 °C) [15], bacteria-based self-healing concrete has exclusively been shown to work under room temperature freshwater conditions [4,5,6,7,8,9,10,11,12,13,14]. If bacteria-based self-healing concrete is to be realized under low-temperature marine conditions, then the bacteria-based agents used to generate self-healing concrete will need to function under the same conditions. Moreover, bacterial spores added directly to concrete during mixing have been shown to have limited functionality over time [5]. On account of this, bacteria-based agents have been protected in expanded clay particles [6,7,13], and bacteria in diatomaceous earth [8] and melamine-based microcapsules [9], before being incorporated in cementitious materials. Although these strategies have successfully extended the period over which functional healing could be achieved, they do not actively contribute to the healing capacity of these technologies. More recently, alginate has been proposed as a protective carrier for bacterial spores [11] and for the formation of a bacteria-based bead [16], for self-healing concrete applications. The bacteria-based bead of the later study, which is composed of bacterial spores—a bacterial nutrient source (yeast extract) and mineral precursor compound (magnesium acetate) encapsulated in calcium alginate—swelled upon submersion in a low-temperature (8 °C) simulative marine concrete crack solution, forming a bacteria-activated calcite (CaCO_3_)–alginate composite material. It is envisaged that this bacteria-based bead technology, when incorporated in a cementitious material, imparts it with a superior crack-healing action. Figure 1 shows a schematic of the proposed healing mechanism for a cementitious composite incorporated with the bacteria-based bead technology. In the event of cracking and water ingress (Figure 1a), beads along the crack would swell, clogging the crack (Figure 1b), which would concomitantly “free up” the bacterial spores, the yeast extract and magnesium acetate. This “freeing up” will then lead to: the magnesium of the magnesium acetate precipitating as magnesium-based minerals, the spores germinating as a result of their exposure to the solubilized yeast extract, and their metabolizing of the acetate, inducing calcium-based mineral precipitation in and on the surface of the bacteria-based beads, healing the crack (Figure 1c).

Here, we test the functionality of the bacteria-based bead through oxygen consumption measurements in a low-temperature (8 °C) artificial marine concrete crack solution (AMCCS). We then incorporate the bacteria-based beads into mortar specimens, forming a bacteria-based self-healing cementitious composite. The bacteria-based self-healing cementitious composite is then assessed for its crack-healing capacity and strength development after being submerged in artificial seawater at 8 °C.

## 2. Materials and Methods

### 2.1. Experimental Program

**Figure 2:**
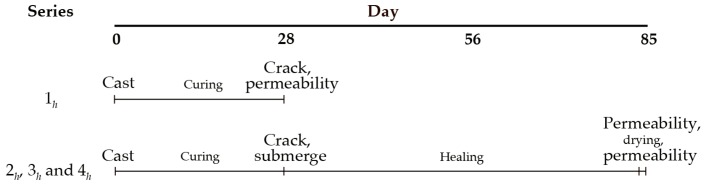
Scheme showing the experimental program used to quantify the crack-healing capacity of the bacteria-based self-healing cementitious composite. The program consisted of four series: (1*_h_*) to establish the initial permeability of unhealed cracked mortar specimens; (2*_h_*) to determine the autogenous healing capacity of cracked mortar specimens after 56 days of submersion in artificial seawater at 8 °C, and the same specimens after drying; (3*_h_*) to determine the autonomous healing capacity of cracked mortar specimens incorporated with beads containing mineral precursor compounds after 56 days of submersion in artificial seawater at 8 °C, and the same specimens after drying; and (4*_h_*) to determine the autonomous healing capacity of cracked bacteria-based self-healing cementitious specimens tested after 56 days of submersion in artificial seawater at 8 °C, and the same specimens after drying. Each series consisted of seven specimens with cracks 0.4 mm and 0.6 mm wide.

The experimental program for the current study consists of two parts. Part 1 assesses the bio-functionality of the bacteria-based bead of Palin et al. [16] under low-temperature marine concrete crack conditions. Three series were set up to help assess the functionality of the bead: (1*_b_*) consisting of plain cement paste specimens submerged in an AMCCS at 8 °C; (2*_b_*) consisting of cement paste specimens embedded with beads containing mineral precursor compounds submerged in an AMCCS at 8 °C; and (3*_b_*) consisting of cement paste specimens embedded with bacteria-based beads submerged in an AMCCS at 8 °C. In Part 2, the bacteria-based bead technology is incorporated in mortar, forming a bacteria-based self-healing cementitious composite. The bacteria-based self-healing cementitious composite is then assessed for its healing capacity and strength development under low-temperature marine conditions. The healing capacity of the composite is tested through its crack water permeability. Four series were set up to help quantify the crack-healing capacity of the bacteria-based cementitious composite: (1*_h_*) to establish reference permeability values for plain mortar specimens with 0.4 and 0.6 mm wide cracks; (2*_h_*) to determine the autogenous crack-healing capacity of plain mortar specimens with 0.4 and 0.6 mm wide cracks following 56 days of submersion in artificial seawater at 8 °C, and the same specimens after drying; (3*_h_*) to determine the autonomous crack-healing capacity of mortar specimens incorporated with beads containing mineral precursor compounds with 0.4 mm and 0.6 mm wide cracks following 56 days of submersion in artificial seawater at 8 °C, and the same specimens after drying; and (4*_h_*) to determine the crack-healing capacity of specimens made from the bacteria-based self-healing cementitious composite with 0.4 mm and 0.6 mm wide cracks following 56 days of submersion in artificial seawater at 8 °C, and the same specimens after drying (Figure 2). The strength development of the bacteria-based cementitious composite is assessed through compressive strength testing. Two series were set up to assess the compressive strength development of the composite: (1*_c_*) to determine the compressive strength of plain mortar cubes, and (2*_c_*) to determine the compressive strength of mortar cubes incorporated with beads containing mineral precursor compounds, submerged in artificial seawater at 8 °C.

### 2.2. Production of the Bacteria-Based Beads

A precursor solution was prepared consisting of sodium alginate (1.5% (*w/v*)) (FMC Health and Nutrition, Philadelphia, PA, USA), magnesium acetate (6.4 g L^−1^; Sigma-Aldrich, St. Louis, MO, USA), yeast extract (0.48 g L^−1^; Sigma-Aldrich), acetic acid (Sigma-Aldrich), and bacteria spores (7 × 10^8^ L^−1^). Acetic acid was used to adjust the pH of the solution to 6 to safeguard against spore germination during bead production. The bacterium, magnesium acetate and yeast extract used in this study are based on the work of Palin et al. [17]. Phylogenetic analysis of the bacterial isolate has revealed it to have a 99% coverage with *Bacillus halmapalus,* its nearest described relative [18]. The precursor solution was pumped drop-wise at a height of 5 cm into a gelling solution of calcium acetate (6.4 g L^−1^) (Sigma-Aldrich). These drops, on entering the calcium acetate solution, cross-polymerized, forming bacteria-based beads (i.e., calcium alginate beads containing bacterial spores, yeast extract and magnesium acetate). These bacteria-based beads, produced in batches, were removed from the calcium acetate solution after 30 min, washed three times with tap water, and dried for 24 h at 36 °C. Beads containing mineral precursor compounds (i.e., calcium alginate beads containing magnesium acetate, yeast extract and no bacterial spores), and calcium alginate beads, were produced as controls. For more information on the production of the beads, see Palin et al. [16].

### 2.3. Bio-Functionality of the Bacteria-Based Bead

**Figure 3:**
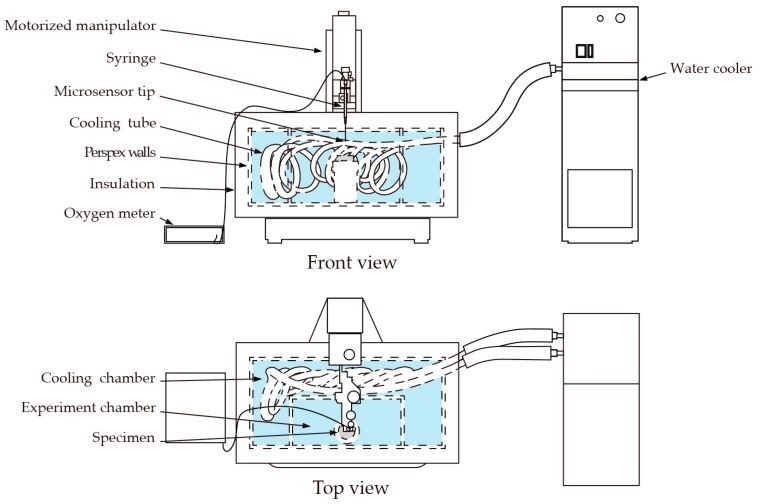
Schematic showing the setup used to test the bio-functionality of the bacteria-based beads. The setup consisted of: an insulated tank, oxygen microsensor, and water cooler. The tank was made up of an experiment chamber containing an artificial marine concrete crack solution (AMCCS) and cooling chamber containing freshwater. A cooling tube attached to a water cooler was used to cool the water of the cooling chamber, which was in turn used to cool the AMCCS of the experiment chamber to 8 °C. Specimens were submerged in the AMCCS of the experiment chamber, and oxygen measurements made with a microsensor mounted to a motorized micromanipulator.

**Figure 4:**
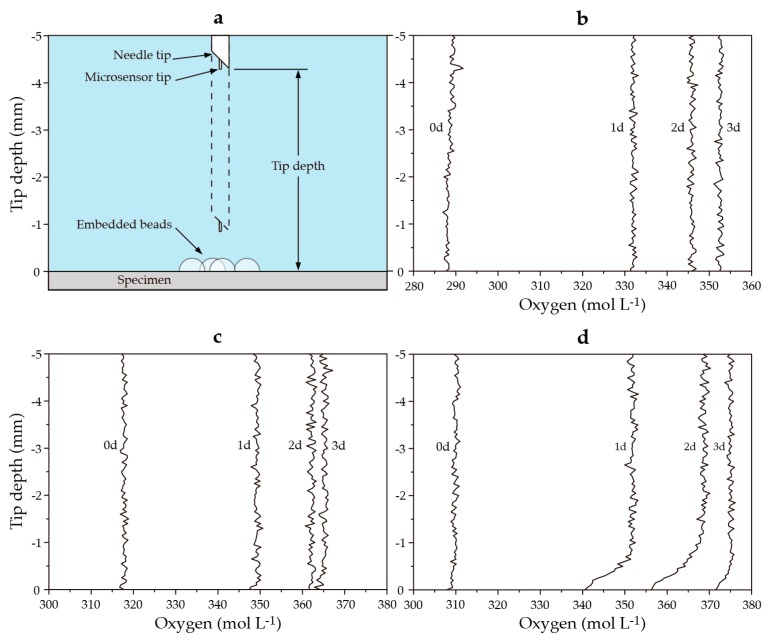
(**a**) A schematic of the water column tested above the specimens; and (**b**–**d**) graphs showing the dissolved oxygen (DO) microprofiles above (**b**) a cement paste specimen; (**c**) a cement paste specimen embedded with beads without bacterial spores; and (**d**) a cement paste specimen embedded with bacteria-based beads, all submerged in an artificial marine concrete crack solution (AMCCS) at 8 °C. The height of the schematic diagram corresponds to the height of the graph profiles. 0d–3d represent profiles taken after zero, one, two, and three days, respectively.

The bacteria used in this study consume oxygen as a result of their metabolic activity. Oxygen depletion, therefore, provides a valuable indicator as to the bio-functionality of the bacteria-based beads. In the current study, we were interested as to whether the bacteria-based beads were able to function in a low-temperature (8 °C) marine concrete crack. To test this, a specially designed setup was devised consisting of an insulated tank, oxygen microsensor, and water cooler (Figure 3). The insulated tank further consisted of an experiment chamber filled with an AMCCS, and a cooling chamber filled with freshwater. The AMCCS was produced by adding crushed mortar pieces to artificial seawater (Table 1), filtering, and adjusting with artificial seawater to a final pH of 9.6. The artificial seawater used is based on the major constituents of natural seawater [19]. A cooling tube attached to a water cooler was used to cool the water of the cooling chamber, which was in turn used to cool the AMCCS of the experiment chamber to 8 °C. Cement disc specimens (10 mm thick and with a diameter of 33.5 mm) were prepared to test the bio-functionality of the bacteria-based beads. Plain cement discs were cast for series 1*_b_*, cement discs embedded with beads containing mineral precursor compounds for series 2*_b_*, and cement discs embedded with bacteria-based beads for series 3*_b_*. Discs were cast from blast furnace slag cement (CEM III/B 42.5 N LH, ENCI, Maastricht, The Netherlands) with a water-to-cement ratio of 0.5. Five beads were embedded in a cluster in the face of the freshly cast discs. The disc specimens were carefully removed from their molds after 24 h, sealed in polyethylene plastic bags, and kept at room temperature for a total curing period of 28 days. Following curing, the discs were submerged in the AMCCS of the experiment chamber. Oxygen concentrations were measured in the water column above the specimens with a fiber-optic oxygen microsensor (NTH-PSt1, PreSens GmbH, Regensburg, Germany) (Figure 4a). The microsensor was mounted to a motorized micromanipulator with a high precision vertical axis (0.1 µm resolution). Oxygen microprofiles were measured in 20 µm steps from 5 mm above the specimens to the surface of the beads. Microsensor measurements were converted to oxygen concentrations via an oxygen meter (Microx TX3, PreSens GmbH). Dissolved oxygen concentrations were monitored over a one-week period. Specimens were tested in duplicate.

### 2.4. Specimen Preparation for Permeability and Compression Testing

Two specimen geometries were produced to test the healing capacity and compressive strength development of the bacteria-based, self-healing cementitious composite. Cylindrical specimens (60 mm long and with a diameter of 33.5 mm) having two diametrically opposite grooves (2 mm wide and 3 mm deep) running down their side were cast for the permeability test; and cube specimens (40 × 40 × 40 mm) were cast for compressive strength testing. Plain mortar cylinders were cast for series 1*_h_* and 2*_h_*, mortar cylinders containing beads with mineral precursor compounds for series 3*_h_*, and cylinders cast from the bacteria-based self-healing cementitious composite for series 4*_h_*. The applied mix design used to produce the bacteria-based cementitious specimens is shown in Table 2. Plain mortar cubes were cast for series 1*_c_*, and mortar cubes incorporated with beads containing mineral precursor compounds were cast for series 2*_c_*. It was decided not to cast mortar cubes containing bacteria-based beads for compressive strength testing, as it was thought that the influence of bacteria for this test would be negligible. Following casting, all specimens were sealed in polyethylene plastic bags and kept at room temperature for a total curing period of 28 days. Following curing, the cylinder specimens were wrapped in polyethylene film and steel rods placed at their grooves. A vise was tightened to the steel rods and each specimen split diametrically from groove to groove. The specimens were carefully unwrapped, and metal gauges 2.4 mm and 2.6 mm thick were fitted between their grooves to achieve defined crack widths of 0.4 mm and 0.6 mm. A two-part adhesive, Plex 7742 and liquid Pleximon 801 (Evonik Röhm GmbH, Essen, Germany), was mixed and applied on either side of the gauges. The gauges were removed and the remainder of the grooves glued. More information on the process taken to prepare the cylinders can be found in Palin et al. [20].

### 2.5. Crack Permeability Test

Cylinder specimens from series 1*_h_* were first assessed for their permeability. Specimens were placed in permeability cells and the permeability cells were attached via fittings to the bottom of the permeability columns. Artificial seawater was prepared and poured into reservoirs at the top of each column. Taps in each reservoir were released, initiating the permeability test. The permeability test was run for 10 min, and any water flowing from the cracks was individually collected in catchment buckets and the weight of the water recorded. The water level of each column was manually maintained between 1 and 1.05 m, giving an almost constant water head of 0.1 bar. Cylinder specimens of series 2*_h_*, 3*_h_* and 4*_h_* were submerged in artificial seawater and vacuumed for 2 h to remove any air bubbles trapped in their cracks. Each series was then transferred to a plastic bucket containing artificial seawater (4 L). Lids were placed on the buckets, and the buckets placed in a refrigerator at 8 °C. The artificial seawater in the buckets was changed once a week for a month to mimic in situ conditions and prevent ion depletion. After 56 days submersion, the specimens from series 2*_h_*, 3*_h_* and 4*_h_* were assessed for their permeability. Following permeability testing, these specimens were dried in a drier at 36 °C for 24 h, and again tested for their permeability. Specimens were dried to evaluate the effect of drying on healing. Each series consisted of seven replicates each.

### 2.6. Characterization of the Healing Material

Cylinder specimens with the mean permeability of each series were selected for chemical analysis. These specimens were dried and polished sections prepared. The sections were then analyzed through environmental scanning electron microscopy (ESEM) (Philips XL 30 ESEM, Philips, Amsterdam, The Netherlands) in back-scattered electron (BSE) mode and elemental mapping through energy dispersive spectroscopy (EDS) (Philips EDAX, Philips). In between preparation and analysis, specimens were kept in a desiccator to avoid continued cement hydration. EDS image maps of calcium, magnesium and silica were merged through the apply image tool in Photoshop 6.0 (Adobe Systems, San Francisco, CA, USA).

### 2.7. Strength Development

Cube specimens were placed in buckets in batches of five and submerged in 4 L of artificial seawater. Lids were placed on the buckets, and the buckets placed in a refrigerator at 8 °C. Artificial seawater in the buckets was changed once a week for a month to mimic in situ conditions and prevent ion depletion. Cube specimens from series 1*_c_* and 2*_c_* were removed from their buckets and tested for their compressive strength 2, 7, 28, 84 and 168 days after casting. Compressive strength testing was conducted with a tensile/compression test machine in conjunction with a servo-plus control unit (Matest, Treviolo, Italy) at a rate of 1 MPa s^−1^. Cubes kept in sealed polyethylene plastic bags were also tested 168 days after casting to test for any difference between their compressive strength and the compressive strength of the submerged specimens.

## 3. Results

### 3.1. Bio-Functionality of the Bacteria-Based Bead

Figure 4a shows a schematic of the water column tested above the specimens. Figure 4b–d show graphs of the dissolved oxygen (DO) concentrations in the water columns above: a cement paste specimen (Figure 4b); a cement paste specimen embedded with beads containing mineral precursor compounds (Figure 4c); and a cement paste specimens embedded with bacteria-based beads (Figure 4d), all submerged in an AMCCS at 8 °C. Specimens embedded with bacteria-based beads showed a decrease in DO in the diffusive boundary layer (1 mm above the beads) after one day and reached their lowest relative concentration after two days (Figure 4d). In contrast, DO concentrations in diffusive boundary layers above the plain cement specimens and specimens embedded with beads containing the yeast extract and acetate did not show such a decrease (Figure 4b,c). DO concentrations in the water columns above the specimens did change over time and may be attributable to changes in temperature.

### 3.2. Crack-Healing Capacity

**Figure 5:**
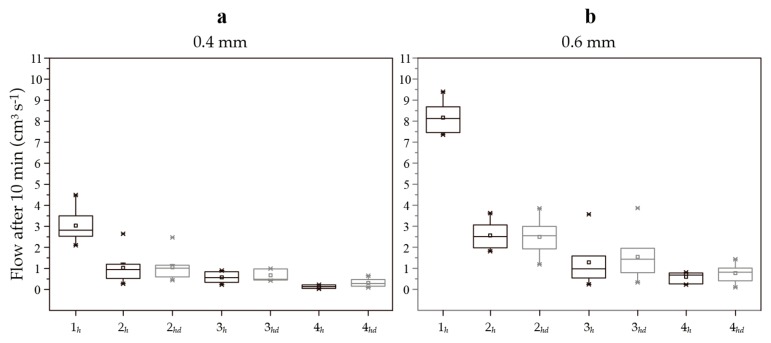
Box plot graphs depicting the permeability data for each series. Graph (**a**) shows the permeability data for specimens with cracks 0.4 mm wide and graph (**b**) shows the permeability data for specimens with cracks 0.6 mm wide. 1*_h_*: Initial permeability for cracked unhealed mortar specimens; 2*_h_*: Permeability of cracked mortar specimens after 56 days of submersion in seawater at 8 °C; 2*_hd_*: Permeability of the 2*_h_* specimens after drying; 3*_h_*: Permeability of cracked mortar specimens with beads containing mineral precursor compounds after 56 days of submersion in seawater at 8 °C; 3*_hd_*: Permeability of the 3*_h_* specimens after drying; 4*_h_*: Permeability of cracked bacteria-based self-healing cementitious specimens after 56 days of submersion in seawater at 8 °C; 4*_hd_*: Permeability of the 4*_h_* specimens after drying. Each box plot represents the permeability data for seven separate specimens. The square symbol of the boxes represents the mean permeability; the whiskers the minimum and maximum permeability values; and the top, middle and bottom lines the 75th, 50th and 25th percentiles, respectively.

Figure 5a shows crack permeability data for specimens with cracks 0.4 mm wide, and Figure 5b shows crack permeability data for specimens with cracks 0.6 mm wide. The mean initial permeability of specimens having cracks 0.4 mm wide was 3.0 cm^3^ s^−1^ (Figure 5a (1*_h_*)). Specimens made from the bacteria-based self-healing cementitious composite with cracks 0.4 mm wide had the lowest permeability of 0.1 cm^3^ s^−1^ after 56 days of submersion (i.e., 5% of the initial permeability) (Figure 5a (4*_h_*)). The mean initial permeability of specimens with cracks 0.6 mm wide was 8.2 cm^3^ s^−1^ (Figure 4b (1*_h_*)). Specimens made with the bacteria-based self-healing cementitious composite with cracks 0.6 mm wide had the lowest permeability of 0.7 cm^3^ s^−1^ after 56 days of submersion (i.e., 7% of the initial permeability) (Figure 5b (4*_h_*)). Specimens containing beads had higher crack permeabilities following drying, while plain mortar specimens had similar permeabilities before and after drying. Further, the permeability data produced by the bacteria-based specimens displayed a lower standard deviation (SD) than the other specimens following submersion.

### 3.3. Healing within the Cracks

**Figure 6:**
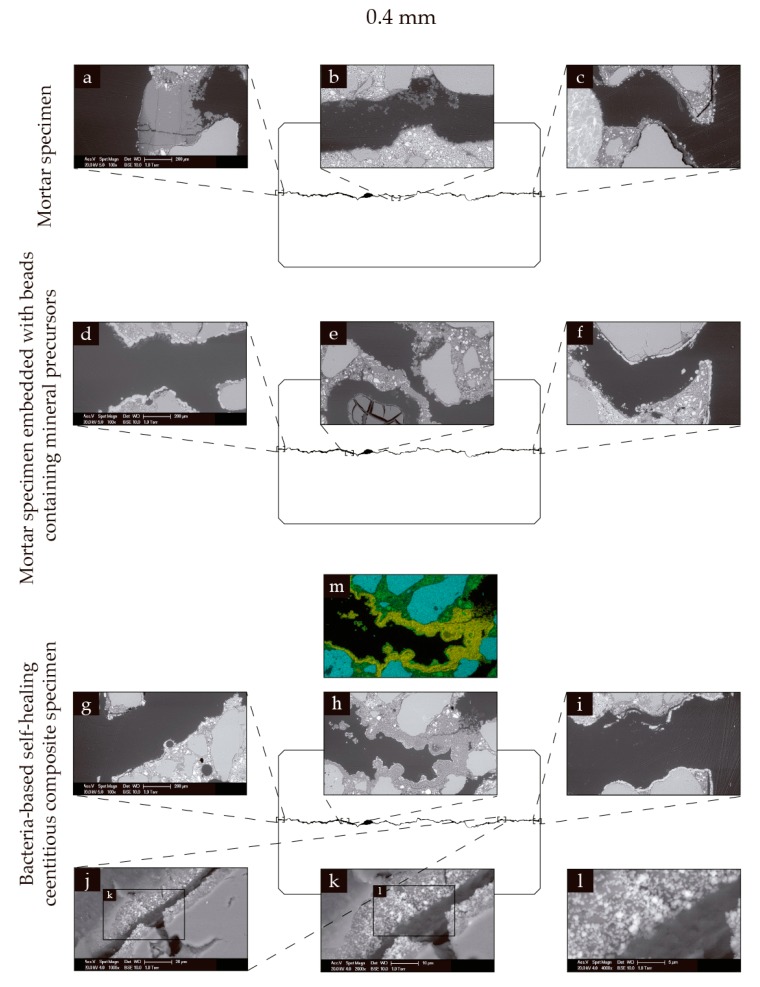
Environmental scanning electron microscopy (ESEM) and energy dispersive spectroscopy (EDS) images of specimens with 0.4 mm wide cracks. (**a**–**c**) ESEM images of a mortar specimen following 56 days of submersion in seawater at 8 °C: (**a**) a crack mouth; (**b**) an area towards the center of the crack; and (**c**) the other crack mouth. (**d**–**f**) ESEM images of a mortar specimen incorporated with beads containing mineral precursor compounds after 56 days of submersion in seawater at 8 °C: (**d**) a crack mouth; (**e**) an area towards the center of the crack; and (**f**) the other crack mouth. (**g**–**l**) ESEM images of a bacteria-based self-healing cementitious composite specimen following 56 days of submersion in seawater at 8 °C: (**g**) a crack mouth; (**h**) an area towards the centre of the crack; (**i**) the other crack mouth; (**j**) a section along the crack of a bacteria-based bead; and (**k**,**l**) successive close-ups of the bead. The EDS elemental map (**m**) corresponding to ESEM image (**h**). Green in the EDS maps represents calcium, yellow represents magnesium, and blue represents silicate.

In order to assess healing within the cracks, polished sections were prepared of selected specimens. These polished sections were then analyzed through ESEM and EDS. Figure 6a–c shows ESEM images of a mortar specimen with a 0.4 mm wide crack following 56 days of submersion in seawater at 8 °C, and subsequent drying. Dark grey precipitates (binary image) could be seen to block one of the crack mouths (Figure 6a), and dark grey formations could also be seen towards the center of the crack (Figure 6b). Figure 6d–f shows ESEM images of a mortar specimen incorporated with beads containing mineral precursor compounds following 56 days of submersion in seawater at 8 °C, and subsequent drying. Limited precipitation could be seen at the crack mouths (Figure 6d,f). Dark grey precipitates could be seen along the crack (Figure 6e). Figure 6g–l shows ESEM images of a specimen made from the bacteria-based self-healing cementitious composite with a 0.4 mm wide crack after 56 days of submersion in seawater, and subsequent drying. Again, limited precipitation could be seen at the crack mouths (Figure 6g,i). Dark grey precipitates could be seen towards the center of the crack, and to fully bridge the crack (Figure 6h). Figure 6m shows the EDS elemental map corresponding to Figure 6h, revealing the dark precipitates to be magnesium-based. White precipitates could also be seen in and on the surface of the bacteria-based beads (Figure 6j–l). EDS elemental mapping revealed these precipitates to be calcium-based.

**Figure 7:**
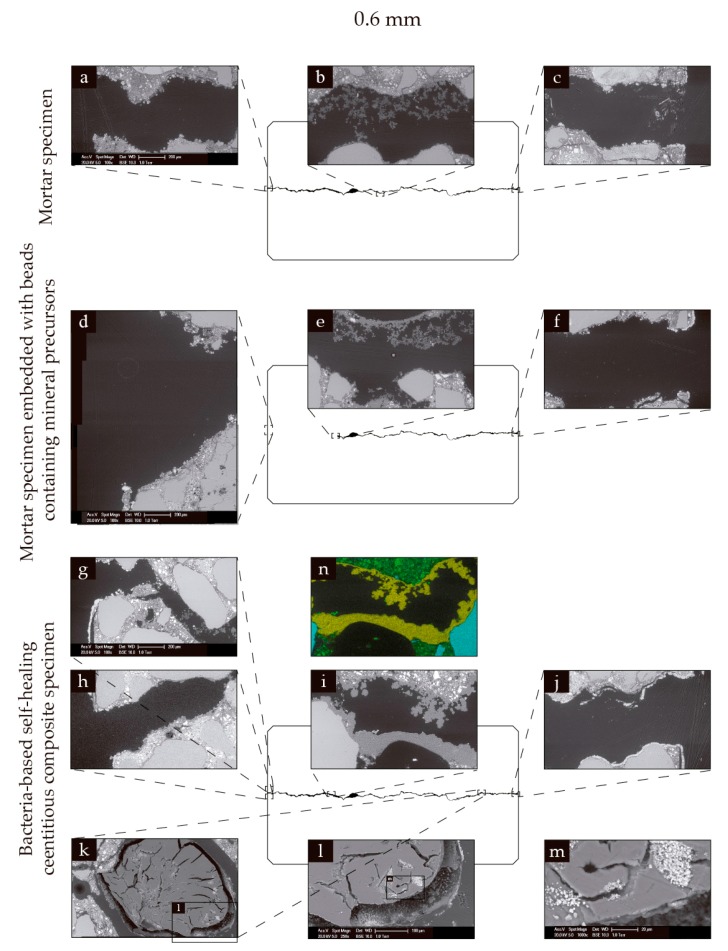
Environmental scanning electron microscopy (ESEM) and energy dispersive spectroscopy (EDS) images of specimens with 0.6 mm wide cracks. (**a**–**c**) ESEM images of a mortar specimen following 56 days of submersion in seawater at 8 °C: (**a**) a crack mouth; (**b**) an area towards the center of the crack; and (**c**) the other crack mouth. (**d**–**f**) ESEM images of a mortar specimen incorporated with beads containing mineral precursor compounds after 56 days of submersion in seawater at 8 °C: (**d**) a crack mouth; (**e**) an area towards the centre of the crack; and (**f**) the other crack mouth. (**g**–**m**) ESEM images of a bacteria-based self-healing cementitious composite specimen following 56 days of submersion in seawater at 8 °C: (**g**,**h**) a crack mouth, which had split into two smaller cracks; (**i**) an area towards the centre of the crack; (**j**) the other crack mouth; (**k**) a section along the crack including a bacteria-based bead; and successive close-ups (**l**, **m**) of a bacteria-based bead. The EDS elemental map (**n**) corresponding to ESEM image (**i**). Green of the EDS maps represents calcium, yellow represents magnesium and blue represents silicate.

Similar phenomena could be seen for specimens with 0.6 mm wide cracks. Figure 7a–c show ESEM images of a mortar specimen with a 0.6 mm wide crack following 56 days of submersion in seawater at 8 °C, and subsequent drying. Limited precipitate formation could be seen at the crack mouths (Figure 7a,c), and dark grey precipitates could be seen towards the center of the crack, reducing its crack volume (Figure 7b). Figure 7d–f show ESEM images of a mortar specimen with a 0.6 mm wide crack incorporated with beads containing mineral precursor compounds following 56 days of submersion in seawater at 8 °C, and subsequent drying. Again, limited precipitation could be seen at the crack mouths (Figure 7d,f), and dark grey precipitates could again be seen towards the center of the crack, reducing the crack volume. Some of these precipitates could be seen to have formed into arc shapes, likely forming on the surface of swollen beads (Figure 7e). Figure 7g–m show ESEM images of a bacteria-based self-healing cementitious specimen with a 0.6 mm wide crack following 56 days of submersion in seawater at 8 °C, and subsequent drying. Limited precipitates could be seen at the crack mouths (Figure 7g,h,j). Dark precipitates were again formed towards the center of the crack, and again in arc-shaped formations (Figure 7i). Figure 7n shows the elemental map corresponding to Figure 7i, which again revealed the dark precipitates to be magnesium-based. White precipitates could also be seen in and on the surface of the bacteria-based beads (Figure 7k–m). EDS mapping again revealed these precipitates to be calcium-based.

### 3.4. Strength Development

**Figure 8:**
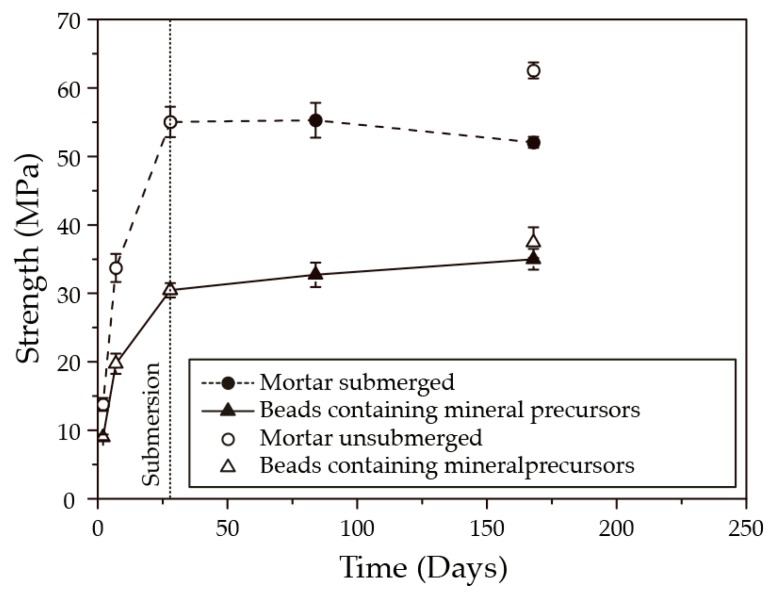
Compressive strength development of mortar cubes and mortar cubes incorporated with beads containing mineral precursor compounds, cured for 28 days and subsequently submerged in artificial seawater at 8 °C.

Figure 8 shows the strength development of both mortar cubes and mortar cubes incorporated with beads containing mineral precursor compounds. Mortar cubes incorporated with beads containing mineral precursors had weaker compressive strengths than the plain mortar specimens. At day 28, the mean compressive strength of the mortar cubes incorporated with the beads was 30.5 MPa (i.e., 55% of the plain mortar cubes). This difference was reduced following 140 days of submersion in seawater at 8 °C, as the compressive strength of the mortar cubes incorporated with beads was 35 MPa, (i.e., 77% of the mortar cubes). The compressive strength of the unsubmerged and submerged mortar cubes incorporated with beads were similar, while the compressive strength of the unsubmerged mortar cubes was higher than the submerged cubes, after 168 days.

## 4. Discussion

A bacteria-based self-healing cementitious composite has been presented for application in low-temperature marine environments. The composite displayed an excellent crack-healing capacity, reducing the permeability of cracks 0.4 mm wide by 95%, and cracks 0.6 mm wide by 93%, following 56 days of submersion in artificial seawater at 8 °C (Figure 5). Healing is attributable to four main mechanisms: mineral precipitation as a result of chemical interactions between the cement paste and seawater; bead swelling; magnesium-based precipitates as a result of chemical interactions between magnesium from the beads and hydroxide ions from the cement paste; and bacteria-based mineral precipitation.

Cementitious materials have an autogenous capacity to heal cracks [21,22,23,24], which is somewhat higher in marine environments [24]. Cracks in cementitious materials allow seawater to come into contact with the cement paste of the crack walls. Since calcium hydroxide is soluble in seawater [25], it leached from the crack walls into the crack solution, whereby the hydroxide ions react with magnesium ions in the seawater, forming magnesium-based mineral precipitates. At the same time, alkalis from the cement paste raise the pH of the crack solution, converting bicarbonate ions in the seawater to carbonate ions. These carbonate ions then react with calcium ions in the crack solution, forming calcium-based mineral precipitates [20,24]. These mineral precipitates give the bacteria-based self-healing cementitious composite a certain autogenous crack-healing capacity. The extent of this capacity can be gauged as the crack-healing capacity of the plain mortar specimens submerged in artificial seawater (series 2*_h_*), which were able to reduce the permeability of cracks 0.4 mm wide by 66%, and cracks 0.6 mm wide by 68%.

Bead swelling and magnesium from the beads give the bacteria-based self-healing cementitious composite a certain crack-healing capacity. Calcium alginate swells in water, meaning that water entering a crack can cause beads along the crack to swell, potentially bridging the crack. This swelling likely “frees up” the magnesium acetate in the beads, making more magnesium available for reaction, with hydroxide ions leached from the cement paste. This magnesium, like the magnesium from the seawater, will then be able to react with hydroxide ions from the cement paste, forming magnesium-based mineral precipitates. These swollen beads also have the potential to provide a support structure upon which mineral precipitates can form. Evidence of bead swelling and the formation mineral precipitates on the surface of swollen beads could be seen in the 0.6 mm wide cracks of specimens incorporated with beads containing mineral precursor compounds (Figure 7e), and specimens made from the bacteria-based self-healing cementitious composite (Figure 7i). The extent of healing provided by bead swelling and magnesium from the beads can be gauged as the difference in the mean permeability of the plain mortar specimens (series 2*_h_*), and the specimens incorporated with beads containing mineral precursor compounds (series 3*_h_*), which equates to 16% for the 0.4 mm wide cracks, and 15% for the 0.6 mm wide cracks (Figure 5).

Bacterial activity also gives the bacteria-based self-healing cementitious composite a certain crack-healing capacity. Swelling of the beads likely “frees up” the bacterial spores, bacteria nutrient source (yeast extract), and mineral precursor compound (acetate) in the beads. This “freeing up” means that the bacterial spores have access to the nutrient source, which causes them to germinate into vegetative cells. The vegetative cells then metabolize the precursor compound, producing carbon dioxide. This carbon dioxide then reacts with calcium ions in and at the surface of the beads, forming calcium-based mineral precipitates. Evidence of these precipitates could be seen in and on the surface of the bacteria-based beads, forming an organic–inorganic composite-healing material (Figure 6j–l and Figure 7k–m). The extent of this capacity can be gauged as the difference between the mean crack permeability for mortar specimens incorporated with beads containing mineral precursor compounds (series 3*_h_*), and specimens made from the bacteria-based self-healing cementitious composite (series 4*_h_*), which equates to 13% for the 0.4 mm wide cracks and 10% for the 0.6 mm wide cracks (Figure 5). The healing attributable to bacteria-induced mineral precipitation was relatively less as compared to chemical precipitation and bead swelling. The bacteria-induced precipitates were, however, a valuable addition, as the bacteria-based cementitious specimens were less affected by drying than the beads without bacteria. The bacteria-based cementitious specimens also produced permeability data with a lower SD than the other two series, attesting to the technologies reliability (Figure 4). Further, this may not represent the full extent of bacteria-based healing if, for example, the bacteria had not used up all the yeast extract/acetate. The best way to confirm this would be to test an extra set of samples over a longer period of time. Production and testing of such a set were not feasible as part of the current study.

What is interesting is that the crack-healing capacity was relatively higher for the larger crack width (i.e., the bacteria-based self-healing cementitious composite was able to reduce the permeability cracks 0.4 mm wide by 95%, and cracks 0.6 mm wide by 93%, following 56 days of submersion in artificial seawater at 8 °C). The ability to heal relatively larger crack widths may be due to the beads being able to swell to both crack widths, and its corollary of “freeing up” more bacteria and mineral precursor compounds for chemical and bacteria-induced mineral precipitation. This being the case, the beads may be able to swell still further, allowing the healing of larger crack widths. In fact, in a previous study, the bacteria-based beads were shown to swell to a diameter of 3 mm [16], and so the beads may be able to heal cracks ~3 mm wide. Drying the bacteria-based self-healing cementitious composite resulted in higher crack permeability values (Figure 5). These higher values can in fact be expected, as the swollen beads in the cracks likely contract as a result of drying. This difference was, however, relatively smaller than the beads without bacteria, which might be due to the formation of mineral precipitates in and on the surface of the beads, whose volume is unaffected by drying.

Although the bacteria-based cementitious composite displayed an excellent crack-healing capacity, mortar specimens incorporated with beads demonstrated lower compressive strengths than plain mortar specimens, following 140 days of submersion in seawater at 8 °C (Figure 8). Such trade-offs are well known in nature, whereby the improvement of one function can lead to the diminishing of another [26]. Despite this, the compressive strength of the current bacteria-based cementitious composite is higher than those reported in the literature [13], and strong enough to be considered as a reliable construction material. Such reductions in compressive strength can be expected, as the beads being much softer than the cement matrix act like a kind of porosity. If we assume that the density of the calcium alginate is 1 kg m^−3^ [27], then the beads of the bacteria-based cementitious composite would represent 5% of the materials’ volume. If this 5% volume were air, then we could expect a ~25% reduction in compressive strength as compared with a specimen containing no air [28], which is somewhat in line with the 45% reduction demonstrated by specimens containing the beads (Figure 8). This being the case, the 28-day compressive strength of the bacteria-based self-healing composite could be increased by reducing the amount of beads, or by using a polymer with a higher compressive strength.

In conclusion, the bacteria-based self-healing cementitious composite shows great potential for realizing cost-effective self-healing concrete in low-temperature marine environments, while the formation of an organic–inorganic composite healing material represents an exciting avenue for self-healing concrete research. 

## Figures and Tables

**Table 1:** Composition of the artificial seawater based on the major constituents of natural seawater [19].

Compound	Amount (g L^−1^)
NaHCO_3_	0.19
CaCl_2_·2H_2_O	1.47
MgCl_2_·6H_2_O	10.57
Na_2_SO_4_·10H_2_O	9.02
KCl	0.75
NaCl	24.08

**Table 2:** Mix-design for the bacteria-based self-healing cementitious composite.

Constituent	Amount (kg m^−3^)
Cement (CEM III/B 42.5 N LH)	494
Water	247
Water cement ratio	0.5
Sand fraction (mm):	
1–2	608
0.5–1	426
0.25–0.5	167
0.125–0.25	319
Bacteria-based beads	50
